# Page Kidney Secondary to Large Splenic Artery Aneurysm Bleeding and Its Management by Angioembolization

**DOI:** 10.5812/numonthly.17144

**Published:** 2014-05-04

**Authors:** Santosh Kumar, Kumar Jayant, Sriharsha AS, Sharwan Kumar Singh, Swati Agrawal

**Affiliations:** 1 Department of Urology, Postgraduate Institute of Medical Education and Research, Chandigarh, India; 2 Department of Obstetrics and Gynecology, Medical College Udaipur, Rajasthan, India

**Keywords:** Embolization, Therapeutic, Kidney, Spleen, Aneurysm

## Abstract

**Introduction:**

The Page kidney is a rare phenomenon that refers to hypertension resulting from any external compression of a kidney by a hematoma, tumor, lymphocele, or urinoma. The activation of the renin-angiotensin-aldosterone system is believed to be the main mechanism responsible for development of resistant hypertension in Page kidney.

**Case Presentation:**

We reported a patient with chronic pancreatitis who presented with hypotension due to splenic artery aneurysmal bleed; following the resuscitation, accelerated hypertension secondary to Page kidney caused by perinephric hematoma presented. Early diagnosis by contrast-enhanced computed tomography of the abdomen and renal angiogram was followed by therapeutic angioembolization. However, ultrasound guided aspiration was not done because of denial by the patient for further treatment. Follow-up showed normalization of blood pressure and resolution of hematoma on subsequent abdomen ultrasound evaluation.

**Discussion:**

Splenic artery aneurysm is a very uncommon cause of Page kidney and to our knowledge, it was the first case of its kind ever reported in the literature.

## 1. Introduction

The Page kidney is a rare condition that occurs due to any external compression of kidney by a hematoma, lymphocele, tumor, or urinoma leading to hypertension (HTN). This high-renin state HTN occurs due to activation of the renin-angiotensin-aldosterone system (RAAS) induced by renal hypoperfusion and ischemia (1). Most common causes attributed to the development of perinephric hematoma are traumatic or iatrogenic (e.g. renal biopsy, lumbar block, etc.). Other mentioned associations of Page kidney are renal tumor, renal cyst, vasculitis, and anticoagulation by coumarin. The interval between initial insult and the development of HTN can be as short as few days to as long as few years and may even present with hypertensive emergencies. We reported a case of hypotension due to the bleeding from splenic artery aneurysm followed by the accelerated HTN postresuscitation secondary to Page kidney. The underlying cause was a hematoma formed by aneurysmal bleeding in a patient with chronic pancreatitis who was treated by angioembolization. Patient was further followed on outpatient basis, which showed normalization of blood pressure. Subsequent ultrasound abdomen revealed resolution of hematoma.

## 2. Case Presentation

**Figure 1.**
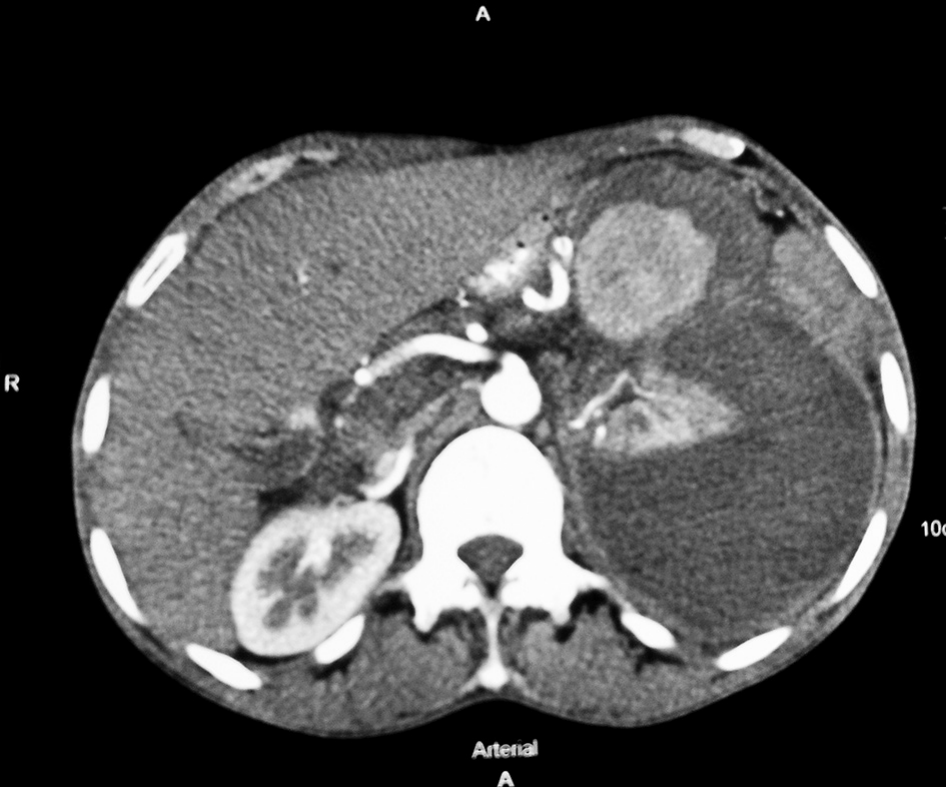
Contrast-Enhanced Computed Tomography of Abdomen Showing Large Splenic Aneurysm With Large Perinephric Hematoma Compressing the Kidney

A 40-year-old male with a medical history of alcoholic liver disease and chronic pancreatitis attended at our emergency department with acute onset of hypotension and severe abdominal pain in epigastrium that was radiating towards back and was associated with nausea and vomiting. He had no history of HTN. On evaluation, he had anemia (Hb, 7 mg/dL), with normal findings on renal function tests (serum creatinine, 0.9 mg/dL; urea, 21 mg/dL). Serum electrolyte assessment showed persistent hypokalemia (serum K^+^, 2.6-3.4 mmol/L) while serum magnesium was normal (serum Mg^2+^, 1.45 mmol/L). Ultrasonography of the whole abdomen showed left perinephric hematoma with normal-sized left kidney (11 cm); contralateral kidney was also normal. Subsequently, patient underwent contrast-enhanced computed tomography (CECT) of the abdomen with computed tomography angiography which showed large perinephric hematoma measuring 16.8 × 14.7 cm, secondary to splenic artery aneurysmal bleeding (Figure 1). There were no findings suggestive of renal artery stenosis on color Doppler ultrasound assessment. Plasma renin activity was 60 ng/mL/h (normal range, 0.2-2.8 ng/mL/h) with a plasma aldosterone level of 46.3 ng/dL.

**Figure 2.**
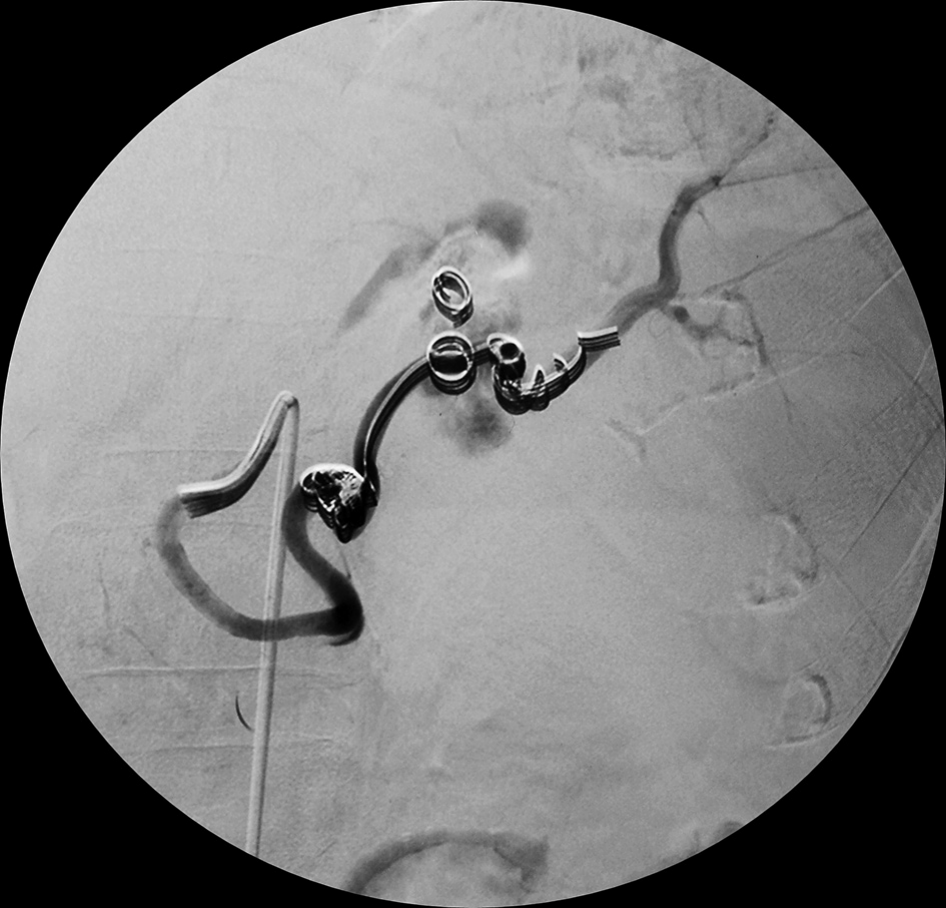
Digital Subtraction Angiography Depicting Angioembolization of the Splenic Artery Aneurysm

**Figure 3.**
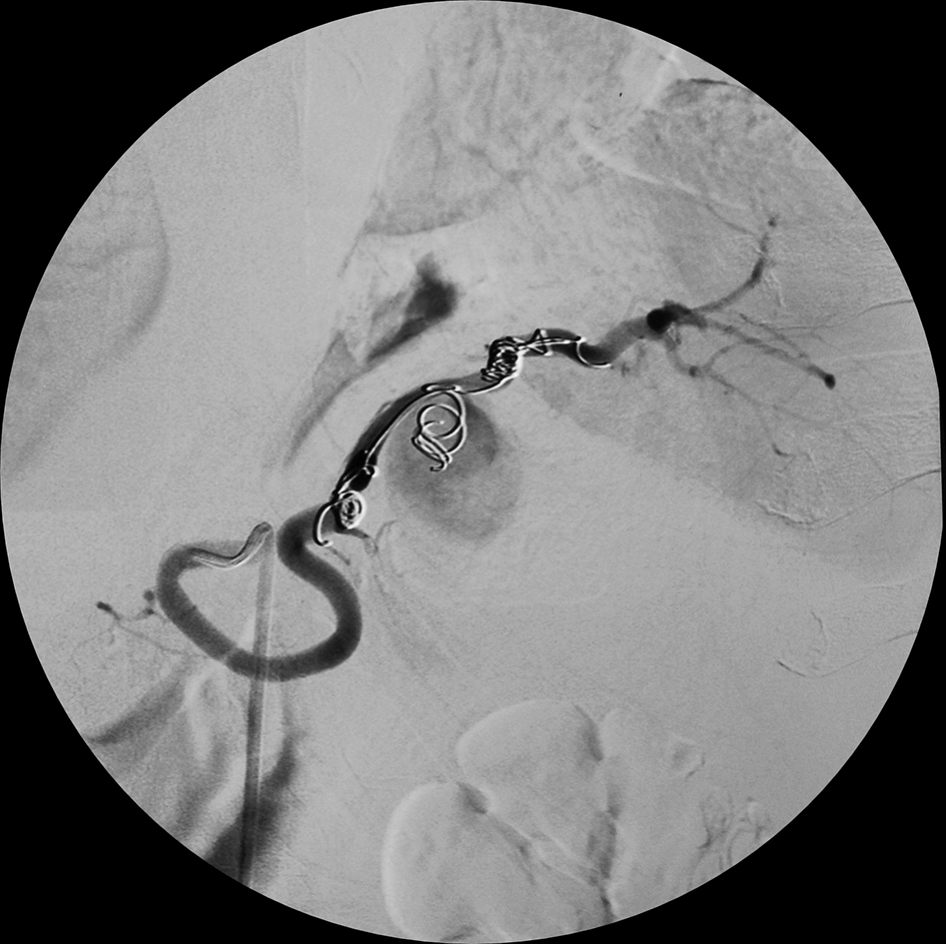
Digital Subtraction Angiography Picture Depicting Resolved Aneurysm After Embolization

After early assessment of patient’s general condition, he was immediately resuscitated with intravenous fluids and three units of packed red blood cell transfusion, along with correction of hypokalemia under ECG monitoring. After initial resuscitation and stabilization, he was taken for angioembolization with coils that led to resolution of aneurysm (Figures 2 and 3). Postprocedure, patient was kept under monitoring. His vital signs remained stable with the hemoglobin level of 11 mg/dL. The next day he developed accelerated HTN. Patient required a combination of antihypertensive drugs for its treatment. The patient refused any further surgical or percutaneous intervention although he was informed regarding the disease process and its consequences. He was discharged on medication.

## 3. Discussion

Page kidney was initially described by Page in 1939 (2). The main mechanism responsible for emergence of accelerated HTN and hypokalemia is believed to be RAAS-mediated. Although Page kidney seems to be similar to the Goldblatt model of renovascular HTN, the latter is due to compression or stenosis of major renal vessels. On the other hand, Page kidney seems to be the result of an ischemic renal vascular insult. Up to this date, approximately 110 cases are reported in literature (3). Most cases are due to compression of the kidney by a subcapsular or perinephric hematoma. Earlier blunt abdominal trauma was the major cause of this hematoma although nowadays many cases (ten out of 28 cases) are iatrogenic injuries following renal transplant biopsy of the kidney (4). In our case, the reason behind hypotension was bleeding from splenic artery aneurysm, which evolved as a complication to chronic pancreatitis, while the development of accelerated HTN was due to the perinephric hematoma. However, the case we reported here is the first of its kind. Up to this date, no case of Page kidney consequent to splenic artery aneurysm has been reported. Pathological Page kidney is seen in acute and chronic types. The acute type is due to the collection of blood or fluid in the subcapsular or perinephric space, which is often short lived, and the chronic type has a delayed onset and is caused by a fibrocollagenous scar compressing the renal parenchyma. Renal insufficiency is not usually seen because of normal perfusion of contralateral kidney, which maintains normal renal function. With time, renal function may gradually decline due to progressive shrinkage of the affected kidney. The development of HTN and deterioration of the renal function probably occurs due to the compression-induced interstitial nephritis. However, in cases of renal allograft or solitary kidney, renal insufficiency may need urgent surgical intervention. Page kidney can be diagnosed by multiple imaging techniques with each modality having its own advantages and disadvantages. Ultrasound is easily available, cheap, and noninvasive; however, small perinephric hematomas can be missed due to its high operator dependence. By assessing the vascularity, color Doppler ultrasound can be helpful not only in diagnosing Page kidney but also in excluding renal artery stenosis. Abdominal CECT is noninvasive and has high sensitivity and specificity in diagnosis of very small hematomas; hence, it has become the modality of choice. Magnetic resonance angiography is helpful in evaluating the age of hematomas and patency of renal vessels. Other investigations such as intravenous urography (IVU), nuclear scan, and renal arteriography also play a role in diagnosing perinephric hematoma.

The initial treatment usually involves drugs such as angiotensin convertase enzyme inhibitors (ACEIs) or aldosterone receptor blocker (ARB) with the aim of normalizing high blood pressure. The inability of a single agent to control the blood pressure necessitates the combination of multiple drugs such as beta blockers and calcium channel blockers (5). Earlier definitive treatment of Page kidney involved radical nephrectomy or open surgery to evacuate the hematoma or performing a decapsulating procedure to save the normal kidney (6, 7). The current approach is inclined toward less invasive procedures such as angioembolization and percutaneous drainage.

To summarize, Page kidney is a rare cause of HTN secondary to RAAS activation. Although splenic artery aneurysm due to chronic pancreatitis is a very uncommon cause of Page kidney, early evaluation by CECT of abdomen and renal angiogram are diagnostic and less invasive procedures such as angioembolization and percutaneous drainage promise favorable outcome.
